# Spatiotemporal Changes in Transcriptome of Odontogenic and Non-odontogenic Regions in the Dental Arch of *Mus musculus*

**DOI:** 10.3389/fcell.2021.723326

**Published:** 2021-10-14

**Authors:** Dong-Joon Lee, Hyun-Yi Kim, Seung-Jun Lee, Han-Sung Jung

**Affiliations:** ^1^Division in Anatomy and Developmental Biology, Department of Oral Biology, Taste Research Center, Oral Science Research Center, BK21 FOUR Project, Yonsei University College of Dentistry, Seoul, South Korea; ^2^NGeneS Inc., Ansan-si, South Korea

**Keywords:** next-generation sequencing, transcriptome, tooth germ, diastema, time series analysis

## Abstract

Over the past 40 years, studies on tooth regeneration have been conducted. These studies comprised two main flows: some focused on epithelial–mesenchymal interaction in the odontogenic region, whereas others focused on creating a supernumerary tooth in the non-odontogenic region. Recently, the scope of the research has moved from conventional gene modification and molecular therapy to genome and transcriptome sequencing analyses. However, these sequencing data have been produced only in the odontogenic region. We provide RNA-Seq data of not only the odontogenic region but also the non-odontogenic region, which loses tooth-forming capacity during development and remains a rudiment. Sequencing data were collected from mouse embryos at three different stages of tooth development. These data will expand our understanding of tooth development and will help in designing developmental and regenerative studies from a new perspective.

## Introduction

“Eating” is the most primitive and indispensable activity in human life. The WHO has announced that healthy teeth ensure proper chewing, and good health is closely related to improving quality of life (Sheiham, 2005). Many instances of missing teeth can be attributed to acquired causes such as dental caries and periodontal disease, as well as congenital edentulous disease, including hypodontia and anodontia, with a high incidence of 1% (Ezoddini et al., 2007). Dental implants have been regarded as the most effective treatment for tooth loss for the past 30 years (Lee et al., 2017), whereas research on creating teeth in the field of regenerative medicine has been ongoing in the past 30 years. With an aging society, the development of preemptive medicine and regenerative medicine are important aspects of national medical strategies to extend healthy life expectancy in many countries (Takahashi et al., 2020).

Tooth regeneration studies are mainly conducted using rodents as an experimental model, and two large flows are identified: first, based on the interaction of the dental epithelium and underlying mesenchyme in the odontogenic (dental forming) region; second, developing teeth in the non-odontogenic (edentulous) region of the dental arch. Unlike humans, mice and rats have one incisor and three molars that are separated by an edentulous region called the diastema on half of the dental arch (Takahashi et al., 2020). Since the review article of Jernvall and Thesleff on the interaction of the epithelium and mesenchyme in tooth development was published (Jernvall and Thesleff, 2000), regeneration research of the tooth-forming region has been based on the first flow. The second trend is based on the rudimentary tooth in diastema. During the early stage of tooth development, tooth rudiments are observed in diastema. They also form epithelial thickening and transient dental buds similar to those observed during the initiation process of tooth development, such as of incisors and molars; however, the subsequent process stops, resulting in the inability to develop into teeth (Peterkova et al., 2006; Prochazka et al., 2010). The second trend originates from the theory of suppressed (rudimentary) organs during evolution presented by Darwin (Darwin and Kebler, 1859) and summarized by Gould (1977) and Hall (1992) and is well supported by the actual molecular data by Chuong and Noveen (1999). Several signaling pathways are known to regulate the continuation of the tooth development process in diastema, and there have been trials to generate teeth by controlling signaling (Tucker et al., 2004; Witter et al., 2005; Klein et al., 2006).

Recently, the scope of tooth regeneration research has moved from conventional gene modification and molecular therapy *in vitro* and *in vivo* to an approach through genome and transcriptome analyses. Several transcriptome analyses of tooth germ development and tissues in permanent growing incisors of rodents have been published within the last 5 years (Seidel et al., 2017; Yang et al., 2017; Sharir et al., 2019; Chiba et al., 2020; Krivanek et al., 2020; Hallikas et al., 2021). These next-generation sequencing (NGS) studies for tooth development are focused on epithelial–mesenchymal interactions and stem cell biology. To date, there is no sequencing database comparing the transcriptomes of diastema and tooth-forming regions.

In this study, we produced a gene expression dataset of diastema and the tooth-forming region (molar) in the mouse mandible. RNA-Seq was performed on three different developmental stages: the early bud stage [embryonic day (E)12.5], early bell stage (E15.5), and late bell stage (E17.5) (Yu and Klein, 2020). The three stages include the time when the tooth bud is formed, when it is differentiated into odontogenic cells that generate the tooth matrix, and when the actual tooth matrix is formed, calcification begins. RNA was extracted from the diastema and molar regions by stage, and all sample gatherings were repeated with three biological replicates. In addition, incisor regions were collected at E12.5. Samples in each set were obtained from 12 to 15 littermate embryos, and for E12.5, 24 to 26 littermate embryos from two mothers were used. Principal component analysis (PCA) and clustering revealed transcriptomic similarities among the three replicates of each spatiotemporal sample. In addition, a time series analysis of three different stages in each region is presented as an example for further reuse of the dataset. Temporal changes in gene expression patterns as regional factors could be understood with this analysis.

The presented RNA-Seq results are the first datasets describing odontogenic and non-odontogenic regions of mouse embryos and provide a fundamental understanding of the spatiotemporal relationship.

## Materials and Methods

### Sample Collection

All experiments were performed according to the guidelines of the Yonsei University Health System, Intramural Animal Care and Use Committee (YUHS-IACUC). YUHS-IACUC complies with the Guide for the care and use of laboratory animals (National Research Council, United States). Moreover, YUHS-IACUC has passed AAALAC International accreditation program since 2003. Mandibular tooth germ and diastema samples were obtained from Hsd:ICR (CD-1^®^) mouse embryos. Hsd:ICR (CD-1^®^) pregnant female mice were purchased from KOATECH (Pyeongtaek, South Korea).

Three different stages of embryos (E12.5, E15.5, and E17.5) were used, and all sample groups were gathered as three biological replicates (named as set1, set2, and set3 in the present study). To obtain sufficient sample amount (for being qualified at initial quality check), samples in each set were obtained from 12 to 15 littermate embryos, and for E12.5, 24 to 26 littermate embryos from two mothers were used.

Embryos of each stage were taken out after the pregnant mice were euthanized. The embryos were placed in a Petri dish with diethyl pyrocarbonate phosphate-buffered saline (DEPC-PBS) at 4°C cold plate.

**FIGURE 1:**
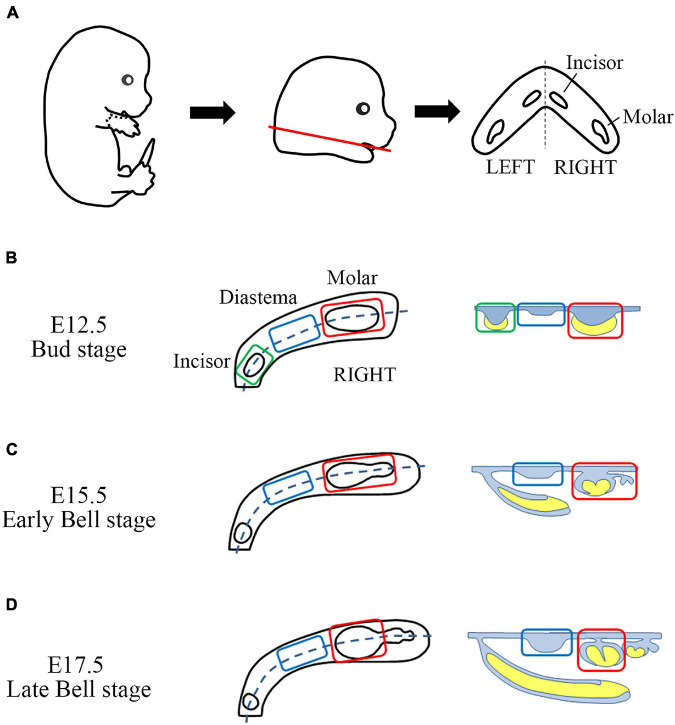
Sample gathering scheme of mouse embryo odontogenic and non-odontogenic regions. **(A)** From E12.5, E15.5, and E17.5 embryos, the craniofacial region was dissected between the first and second branchial arches. Then the mandible was gathered (red line). The tongue was removed. The trimmed mandible has incisor and molar tooth germs as well as diastema regions on both sides. It was halved along the midline. **(B)** From the E12.5 mandible half, the aboral part was removed. The remaining oral part was divided into three parts (incisor, diastema, and molar). **(C,D)** From the E15.5 and E17.5 mandible halves, incisor region, mandibular bone, and forming alveolar bone were removed. The remaining half dental arch composited with the dental epithelium, and the underlying mesenchyme was divided into the diastema and molar regions.

The head of the embryo was cut off under the mandible (between the first and second branchial arches). The cut heads were transferred to a new dish containing DEPC-PBS. The mandible was dissected from the cut head, and the tongue was removed from the mandible. Trimmed mandibles were transferred to a new DEPC-PBS dish and then divided into two halves using 30G 1/2 length injection needles (Figure 1A). For the E12.5 mandible, the aboral part (mandibular bone-forming region, including Meckel’s cartilage) was removed from the oral part. The oral parts were divided into three parts (incisor, diastema, and molar) (Figure 1B). On the stereomicroscope, tooth germs of the incisors and molars can be observed with proper illuminators. For E15.5 and E17.5 mandibles, dental epithelium and underlying mesenchyme of diastema and molar regions were separated from the forming alveolar bone and mandibular bone. They were divided into diastema and molar parts (Figures 1C,D). The tooth germ of the incisor was grown beneath the diastema and molar tooth germ inside the mandibular bone; therefore, it was detached with the mandibular bone.

### RNA Preparation

Dissected samples were collected in Trizol reagent (Invitrogen, CA, United States) and stored at −80°C until RNA extraction. RNA from each set of sample groups was extracted individually according to the instructions of the manufacturer. RNA concentration was assessed using a NanoDrop^TM^ spectrophotometer (Thermo Fisher Scientific, Waltham, MA, United States) to determine the next step. When the total RNA amount of the sample was lower than 3 μg, the sample collection was repeated.

For qualified RNA samples, RNA concentration, total amount, and RNA integrity number (RIN) values were calculated using an Agilent 2100 Bioanalyzer (Agilent Technologies, Santa Clara, CA, United States) (Supplementary Table 1).

### Library Construction and RNA Sequencing

For RNA-Seq analysis, we prepared mRNA sequencing libraries as paired-end reads with a length of 100 bases using the TruSeq Stranded mRNA Sample Preparation Kit (Illumina, San Diego, CA, United States) according to the protocols of the manufacturer. The mRNA molecules were purified and fragmented from 2 μg of total RNA. The libraries were sequenced as paired-end reads (2 × 150 bp) using a NovaSeq 6000 (Illumina).

### Processing of RNA Sequencing Data

#### Filtering

Low-quality reads were filtered according to the following criteria: reads containing more than 10% of skipped bases (marked as “N”s), reads containing more than 40% of bases whose quality scores were less than 20, and reads with average quality scores of less than 20. The quality scores across all bases were calculated using Sanger/Illumina 1.9 encoding. A quality score of 20 indicated a nucleotide accuracy of 99%. The entire filtering process was performed using in-house scripts.

#### Sequence Alignment

Filtered reads were mapped to the reference genome of *M. musculus* using the aligner TopHat (Trapnell et al., 2009) (Illumina).

#### Gene Expression Estimation

Gene expression levels were measured with Cufflinks v2.1.1 (Trapnell et al., 2010) using the gene annotation database of *M. musculus*. To improve the accuracy of the measurement, multi-read-correction and frag-bias-correct options were applied. All other options were set to the default values. Estimation data were displayed as fragments per kilobase of transcripts per million mapped reads (FPKM) and found on Figshare (Lee, 2021b).

#### Data Availability

The RNA-Seq datasets were deposited in the NCBI as a study under accession number SRP308455 (Lee, 2021a). The raw datasets are generated as FASTQ format. A total of 21 raw data can be downloaded from the Sequence Read Archive (SRA). Detailed information on meta-analysis of RNA-Seq has been deposited in Figshare (Lee, 2021b).

#### Differentially Expressed Gene Analysis

Differentially expressed gene (DEG) analysis was performed using “DEGseq” package v1.42.0 in R. To prevent the fold change value of the log scale from being calculated as infinite or non-defined by non-reading (0 value of FPKM) and being excluded as non-significant, all the FPKM values were added 1 E-317, which is less than the smallest value (2.489 E-317) among all gene expression estimations. Genes with fold changes of >2 or <0.5, and *p*-values < 0.05, were defined as upregulated and downregulated DEGs, respectively.

#### Gene Ontology Analysis

To characterize the identified genes from DEG analysis, Gene Ontology (GO) analysis was performed using “clusterProfiler” package v3.16.1 in R v4.0.3. The GO database classifies genes according to the three categories of biological process (BP), cellular component (CC), and molecular function (MF) and provides information on the function of genes. In addition, GO overrepresentation test (Boyle et al., 2004) was performed with the following parameters: GO annotation database: “org.Mm.eg.db (v3.11.4),” *p*-value cutoff: 0.05, *q*-value cutoff: 0.1). All other options were set to the default values.

### Time Series Analysis

For time series analysis, the changes in gene expression level (FPKM) over time were labeled as U (upregulated), F (flat), and D (downregulated) based on log_2_FC = 2.0. The GO summary and GO enrichment results of time series analysis are found on Figshare (Lee, 2021b).

### *In situ* Hybridization

Specimens were fixed overnight in 4% paraformaldehyde in phosphate-buffered saline (PBS). For *in situ* hybridization, the specimens were treated with 20 μg/ml of proteinase K for 6 min (E12.5 mandible) at room temperature. Antisense RNA probes were labeled with digoxigenin (Roche, Switzerland). After *in situ* hybridization, the specimens were frozen sectioned at a thickness of 14 μm. The primer sequences are presented in Supplementary Table 2.

### Real-Time Quantitative Polymerase Chain Reaction

The RNA extracts were reverse transcribed using Maxime RT PreMix (#25081, iNtRON, South Korea). Quantitative polymerase chain reaction (qPCR) was performed using a StepOnePlus Real-Time PCR System (Applied Biosystems, United States). The amplification program consisted of 40 cycles of denaturation at 95°C for 15 s and annealing at 61°C for 30 s. The expression levels of each gene are expressed as normalized ratios against the *Gapdh* housekeeping gene. The primer sequences are presented in Supplementary Table 2.

## Results

### RNA-Seq Library Construction

**FIGURE 2:**
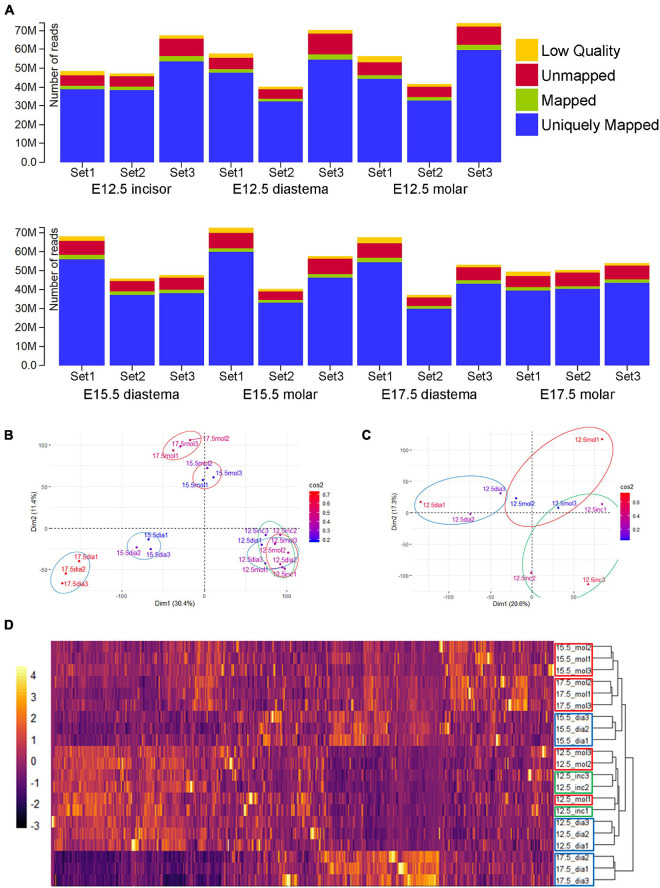
RNA-Seq data alignment and clustering. **(A)** Alignment results of 21 samples. More than 80% of total reads were uniquely mapped (the read mapped to exactly one location within the reference genome). **(B)** Principal component analysis (PCA) result of normalized gene expression (FPKM). E15.5 and E17.5 samples clustered by PC1 and PC2. E12.5 groups seem not separated well. **(C)** PCA result of E12.5 samples. Diastemas were separated from odontogenic regions by the PC1 axis. Incisors and molars were separated by the PC2 axis. **(D)** Heatmap of expression and dendrogram of 21 samples. E12.5 incisors and molar show a similar RNA expression. At E15.5 and E17.5, molars and diastemas had clustered with each other. Particularly, E17.5 diastema showed the most different pattern from other experimental groups.

RNA-Seq libraries were created and sequenced using the Illumina platform. RNA-Seq of 21 samples (seven experimental groups × three biological replicates) was performed and generated as raw data. After obtaining the raw data, the adaptor sequences and low-quality reads were removed (see the “Materials and Methods” section for details). After the quality control process, the filtered reads were mapped to the corresponding genome sequences (Supplementary Table 3). The total number of adapter sequence trimmed of 21 samples was 1,149,147,996 reads, the total number of filtered RNA-Seq reads was 1,110,647,532 (96.65%), and the total mapped RNA-Seq reads were 965,368,578 (84.01% of trimmed raw reads). Read information mapped to each genome and gene is also presented for each sample (Supplementary Table 4). Among the mapped RNA-Seq reads, the reads mapped to exactly one location within the reference genome were identified as “Uniquely Mapped.” The total uniquely mapped RNA-Seq reads were 925,631,629 (80.55% of trimmed raw reads; Figure 2A). Gene expression was normalized and estimated using the FPKM with uniquely mapped RNA-Seq reads.

### Pairwise Correlation Analysis of Biological Replicates and Meta-Information

Pairwise correlation of the gene expression profiles of 21 samples was analyzed (Supplementary Figure 1). All the Pearson correlation coefficients between FPKM of gene expression within each experimental group were more than 0.95, except for E17.5 diastema set1 and set2 [0.936].

Meta-information sets were produced during the sequence alignment. The average coverages of the reads that were aligned to mRNA are shown in pie charts (Supplementary Figure 2). The coverage range was divided by 0.1, from 0 to 1, and the reads number for each range covering the mRNA sequence reference was presented. Reads with a coverage range of 0.9 to 1.0 accounted for 45–55% in approximately all samples.

### Principal Component Analysis (PCA) of Transcriptome

Principal component analysis (PCA) was performed with the gene expression estimation using the “FactoMineR” package v2.4 in R to visualize the transcriptomic similarity among biological replicates and difference among the spatiotemporal samples (Figure 2B). In the E15.5 and E17.5 samples, PCA and clustering revealed transcriptomic similarity among the triplicates. E12.5 samples clustered among themselves, apart from the E15.5 or E17.5 samples. In the PCA analysis of all 21 samples, the difference between the E12.5 odontogenic/non-odontogenic samples was relatively insignificant. For more clear analysis, PCA was performed again with only the E12.5 samples (Figure 2C). Tooth-forming regions (incisor and molar) were separated from the diastema region along the principal component 1 (PC1) axis. The diastema and molar regions were separated from the incisor along the PC2 axis. The gene expression of 21 samples was also presented as a heatmap and dendrogram (Figure 2D) using “pheatmap” package v1.0.12 in R. E12.5, which showed similar expression patterns in incisors and molars, and E17.5 diastema showed the most different pattern from other periods or regions.

### Differentially Expressed Genes and Gene Ontology Term Analysis of Transcriptome Expression

**FIGURE 3:**
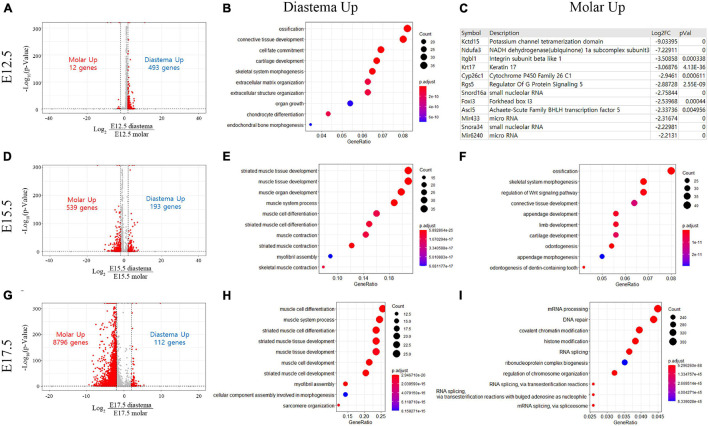
Differentially expressed gene (DEG) and Gene Ontology (GO) analyses of transcriptome in the embryo diastema and molar regions. **(A)** DEG volcano plot of transcriptome in the E12.5 diastema and molar regions. **(B)** The top 10 GO terms associated with increased DEG in diastema. The terms of ossification and tissue developments were displayed. **(C)** Upregulated genes in the E12.5 molar region. **(D)** DEG plot of transcriptome in the E15.5 embryo. **(E,F)** The GO terms most indicated in E15.5 diastema and molar. **(G)** DEG plot of transcriptome in the E17.5 embryo. **(H,I)** GO terms most indicated in the E17.5 diastema and molar.

Differentially expressed gene analysis was performed with FPKM values of transcriptome in three different stages of embryo odontogenic and non-odontogenic regions. DEG results were displayed as volcano plots, and the top 10 GO processes with the largest gene ratios are plotted in order of gene ratio of significantly increased in the diastema and molar regions, respectively. The size of the dots represents the number of genes in the significant DEG list associated with the GO term and the color represents the p-adjusted value. In E12.5, the number of significantly increased genes in the diastema was 493, and 12 genes were increased in the molar (Figure 3A). GO terms associated with increased DEG in the diastema were related to ossification and tissue development (Figure 3B). The number of significantly increased DEG in the molar (Figure 3C) was too small to analyze the GO terms. The transcriptome expression data of E12.5 were presented as a heatmap for each GO term related to tooth development (Supplementary Figure 3). In the E15.5 embryo, DEGs in the molar were increased to 539 genes and decreased in the diastema to 193 genes (Figure 3D). The GO terms most indicated in the E15.5 diastema were related to muscle differentiation (Figure 3E). The Wnt signaling pathway, odontogenesis, and tissue developments of GO terms were indicated in the E15.5 molar (Figure 3F). In the E17.5 embryo, the number of DEGs in the molar region was sharply increased to 8,796 and decreased in the diastema following E15.5 (Figure 3G). GO terms of the diastema in E17.5 were similar to that of E15.5, muscle differentiation (Figure 3H). mRNA processing or RNA splicing-related GO terms were displayed in the E17.5 molar (Figure 3I).

### Time Series Analysis

**FIGURE 4:**
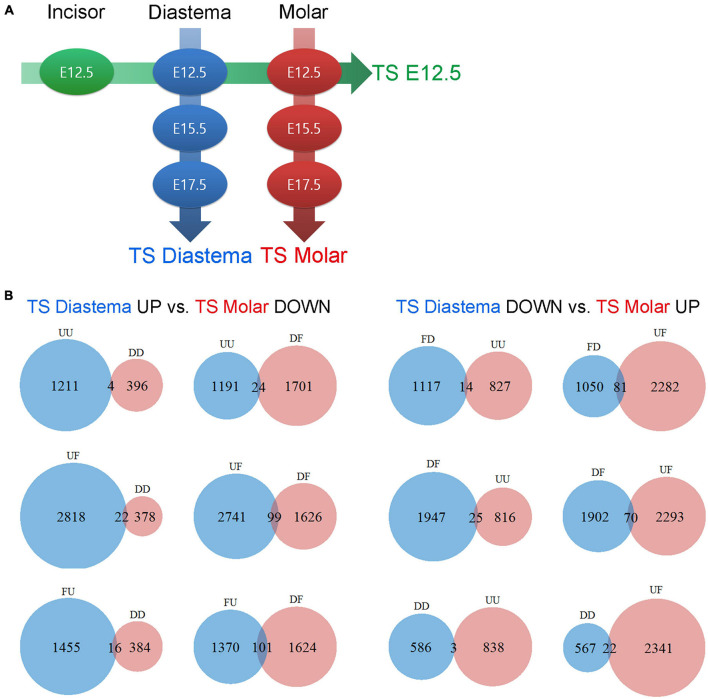
Time series analysis. Time series analysis of time- or region-dependent changes in gene expression patterns. **(A)** Three time series axes were generated from seven experimental groups: TS E12.5, TS Diastema, and TS Molar. **(B)** Gene counts of TS Diastema and TS Molar move in opposite direction in a time-dependent manner. The gene expression patterns between the elements on an axis are labeled as Up (U), Flat (F), or Down (D). UU or DD, genes gradually increased or decreased during development; UF or DF, genes increased or decreased between E12.5 and E15.5, then remained flat between E15.5 and E17.5; FU or FD, genes remained flat between E12.5 and E15.5, and then changed between E15.5 and E17.5.

In this study, gene expression dataset was generated with three different developmental stages of the dental arch. Time series analysis was performed with this dataset. Time series analysis is a method for analyzing time series data in order to extract meaningful statistics and other characteristics of the data. Time series analysis is used to analyze the pattern showing the gene expression levels of three or more time points, which are arranged sequentially. In this case, the sequential arrangement of each time point is called the analysis axis. If the elements of the analysis are set as local factors with the time factor fixed, the sequential arrangement of each location becomes the analysis axis. In this study, three time series analysis axes were set from the datasets provided by seven experimental groups: time series (TS) Diastema, TS Molar, and TS E12.5 (Figure 4A). The gene expression pattern changes according to the developmental stage in the diastema and molar are verified through TS Diastema and TS Molar. With time factor fixed to E12.5, the expression pattern change according to the region on dental arch such as incisor–diastema–molar is verified through TS E12.5.

The gene expression patterns between the elements on an axis are labeled as Up (U), Flat (F), or Down (D). Since three axes set in the present study have three elements, respectively, the analysis result can be presented in nine types of patterns consisting of two letters; UU, UF, UD, FU, FF, FD, DU, DF, or DD. For example, in TS diastema, UF refers to a gene expression pattern that shows an increase as the developmental stage goes from E12.5 to E15.5, and no significant change between E15.5 and E17.5 was found. The GO classification information of the genes corresponding to the nine types of expression patterns shown in the time series analysis is presented (Table 1). The number of genes belonging to the three categories, biological process (BP), cellular component (CC), or molecular function (MF), which constitute the highest hierarchy of GO terms, was organized by expression pattern type. All metrics and detailed time series analysis data including the GO term list can be found on Figshare (Lee, 2021b). The analyses of the diastema and molar regions that move in the opposite direction were presented as Venn diagrams (Figure 4B). The list of oppositely expressed genes over time would show which genes drive odontogenic capacity or keep non-odontogenic characters. Genes relatively upregulated in the diastema and downregulated in the molar region simultaneously were counted to 266. The opposite case was 215.

**TABLE 1:**
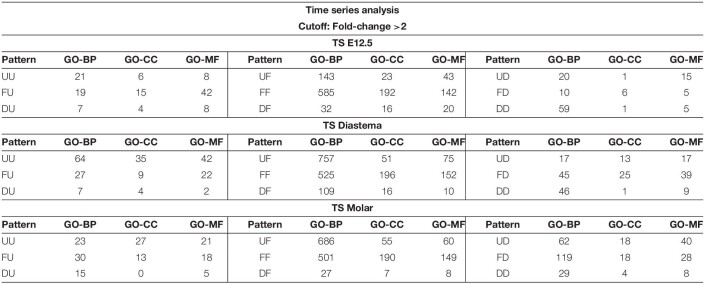
Gene Ontology (GO) classification of time series analysis.

*BP, biological process; CC, cellular component; MF, molecular function.*

The expression profiles of E12.5, the stage to determine whether to form teeth, are composed of sequencing data of two odontogenic regions (incisor and molar) and a diastema region between them. From the DEG analysis, there were just 12 significantly upregulated genes in the E12.5 molar compared with those in the diastema. Therefore, we examined the gene expression that maintains the diastema, not odontogenic capacity, at the E12.5 stage. The UD gene group in TS E12.5 refers to the pattern of upregulated in the diastema and downregulated in the incisor molar region. A list of this gene group is shown in Table 2. The transcriptome of the UD pattern showing a high expression in the diastema was microRNAs, and the biological process category was the most common in GO analysis.

**TABLE 2:** The list of genes which show a UD pattern in time series analysis of TS#12.5 with differentially expressed genes (DEGs) of diastema to molar region.

**Symbol**	Log_2_FC^*^	*p*-value^*^	**Descriptions**	**Symbol**	Log_2_FC^*^	*p*-value^*^	**Descriptions**
Mir7036	33.41712	0	microRNA	Cxcl14	3.267225	4.78E–103	c-x-c motif chemokine ligand 14
Snord35a	32.48927	0	Small nuclear RNA	Mecom	3.258409	1.78E–15	mds1 and evi1 complex locus
Mir6244	31.18008	0	microRNA	Klf14	3.255735	0.000736	Kruppel-like factor 14
Mir6363	31.04732	0	microRNA	Ptprr	3.181666	0.000548	Protein tyrosine phosphatase receptor type R
Gm25640	29.92715	0	Predicted	Scube1	3.176487	2.18E–44	Signal peptide, CUB domain and EGF like domain-containing 1
Snora7a	29.1818	0	Small nuclear RNA	Nr5a2	3.172816	3.23E–12	Nuclear receptor subfamily 5 Group A member2
Mir8112	27.21177	0	microRNA	En1	3.168001	6.92E–05	Engrailed homeobox 1
Mir6376	26.7793	0	microRNA	Tnmd	3.112576	0.000641	Tenomodulin
Snora81	14.20815	0.002464	Small nuclear RNA	Foxc1	3.111953	0.00033	Forkhead box c1
Gm27321	13.46153	0.011719	Zfp408	Phospho1	3.105814	0.026399	Phosphoethanolamine/phosphocholine phosphatase 1
Zfp618	11.64911	0	Zinc finger protein	Col14a1	3.101844	0.001934	Collagen type XIV Alpha1
Ibsp	5.260777	2.20E–10	Integrin-binding sialoprotein	Steap1	3.09431	0.012923	Six transmembrane epithelial antigen of prostate 1
Klk4	4.383986	0.013	Kallikrein-related peptidase 4	Nefm	3.067487	0.005306	Neurofilament medium chain
Reg3g	4.266499	0.000952	Regenerating family member 3 gamma	Rxfp2	3.063933	1.47E–05	Relaxin family peptide receptor2
Gm19705	4.234041	0.018785		Tubb4a	2.99028	7.89E–07	Tubulin beta 4a
Islr2	4.223263	0.000188	Immunoglobulin superfamily containing leucine-rich repeat	Meis1	2.97116	1.49E–11	Meis homeobox 1
Aqp4	4.134305	6.21E–07	Aquaporin 4	Lgr5	2.958614	7.65E–68	Leucine-rich repeat-containing G protein-coupled receptor 5
Zc3h12d	4.132709	0.008292	Zinc finger CCCH type	E130114P18Rik	2.955796	8.45E–07	
Rab17	4.103509	0.000176	Ras-related protein	Lgi1	2.953655	0.018199	Leucine-rich Glioma-inactivated 1
Dgkk	4.090841	0.04119	Diacylglycerol kinase kappa	Gsc	2.922436	3.56E–24	Goosecoid homeobox
Gm10263	3.798916	0.002459		Nfe2	2.861926	0.018314	Nuclear factor, erythroid 2
Slc13a5	3.789301	0.041369	Solute carrier family 13 member 5/sodium-dependent citrate cotransporter	Nfatc2	2.816633	2.09E–05	Nuclear factor of activated T cells
Tbx22	3.752578	2.11E–26	T-Box transcription factor 22	Gpr17	2.781657	0.008879	G protein-coupled receptor 17
Thbs4	3.723435	9.90E–07	Thrombospondin4	Sgk1	2.724953	1.10E–09	Serum/glucocorticoid-regulated kinase 1
Prdm6	3.660177	0.000133	PR/set domain	Tbx15	2.695135	2.38E–20	T-Box transcription factor 15
Gm6030	3.644557	0.003672		Abi3	2.685124	0.004213	ABI family member 3
Nxnl2	3.636729	0.002842	Nucleoredoxin-like 2	Gdf10	2.677306	3.24E–08	Growth differentiation factor 10
Gpr50	3.515921	5.39E–11	G protein-coupled receptor 50	Kera	2.671049	0.004012	Keratocan
Tspan8	3.498695	2.69E–25	Tetraspanin 8	Cdh2	2.64427	3.21E–31	Cadherin 2
Adam23	3.498398	2.75E–15	ADAM metalloproteinase domain 23	Gfra2	2.619614	1.69E–11	GDFN Family Receptor Alpha 2
Pcp2	3.442911	0.000229	Purkinje cell protein 2	Tbx3os1	2.559858	6.35E–49	T-Box transcription factor 3 opposite strand 1
Pappa	3.407946	0.005311	Pappalysin 1	Ccbe1	2.502682	0.048301	Collagen and calcium-binding EGF domains
Itm2a	3.289263	0	Integral membrane protein 2a	Ndp	2.488171	0.028321	Norrin cystine knot growth factor NDP
Fhod3	3.284579	0.001274	Formin Homology 2 domain-containing 3	Gm13547	2.45225	0.044576	
Cldn11	3.28305	5.61E–06	Claudin 11	Col13a1	2.450384	3.22E–06	Collagen type XIII Alpha1
Ly6a	3.274334	0.001522	Lymphocyte antigen 6a	Ccdc3	2.416664	0.00057	Coiled-coil domain-containing 3

*Log_2_FC and *p*-value are DEG analysis of the E12.5 diastema to the E12.5 molar.

### *In situ* Hybridization of Genes Upregulated in Diastema

In order to confirm the results of time series analysis and to identify detailed mRNA expression positions, the five genes were selected among the shown UD pattern in TS E12.5, and *in situ* hybridization of the five genes were performed. To select the genes from Table 2, (1) genes whose expression patterns were already known, (2) genes found to play an important role in the E12.5 stage in tooth formation, and (3) transcriptomes that were not genes (coding for mRNA), such as microRNA, were excluded. Among the genes that passed these criteria, those suitable for *in situ* hybridization probe production were selected: *Zfp618*, *Klk4*, *Gfra2*, *Cxcl14*, *Cdh2*.

**FIGURE 5:**
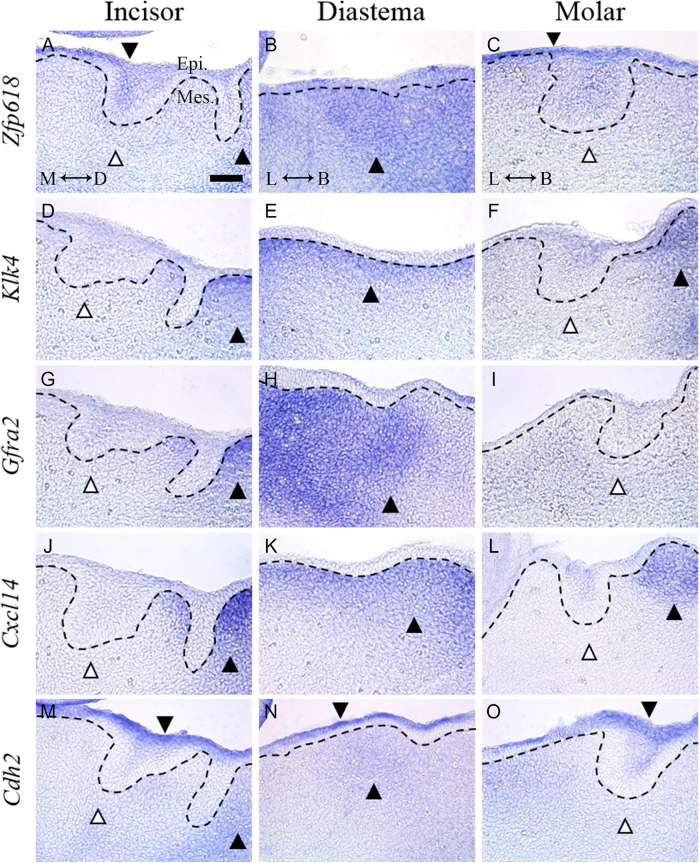
*In situ* hybridization of five genes selected from the DEG list showing UD pattern in TS E12.5. **(A)** *Zfp618* was shown in the oral epithelium and incisor tooth germ epithelium. It was also expressed in the distal side of the vestibular lamina. **(B)** *Zfp618* was expressed in the diastema mesenchyme. **(C)** In the molar, it was weakly expressed in the oral epithelium of the molar region. **(D)** *Klk4* was observed in the distal side of the incisor vestibular lamina **(E)** and diastema mesenchyme beneath the oral epithelium. **(F)** *Klk4* was not expressed in the molar region. **(G)** *Gfra2* was expressed in the distal side of the incisor vestibular lamina. **(H)** It was strongly and broadly expressed in the diastema mesenchyme region. **(I)** In the molar region, *Gfra2* expression was not observed. **(J)** *Cxcl14* expression pattern in the incisor region is similar to *Gfra2* and *Klk4*. **(K)** In the diastema, *Cxcl14* was observed beneath the oral epithelium. **(L)** In the molar region, *Cxcl14* is expressed in the distal side mesenchyme of the molar tooth germ. **(M–O)** *Cdh2* was expressed in the whole oral epithelium of the embryo dental arch. *Cdh2* was also expressed in the diastema mesenchyme; however, it was not expressed in the mesenchyme of odontogenic regions. Dotted lines indicate the basal lamina. Black arrow heads indicate expressed region. White arrow heads indicate not expressed region. M, mesial; D, distal; L, lingual; B, buccal; Epi., epithelium; Mes., mesenchyme; scale bar: 50 μm.

*Zfp618* mRNA was shown in the oral epithelium and incisor tooth germ epithelium. It was also expressed in the mesenchyme of the vestibular lamina distal part (Figure 5A). *Zfp618* was expressed in diastema mesenchyme (Figure 5B). In the molar, it was weakly expressed in the oral epithelium of the molar region (Figure 5C). *Klk4* was observed in the mesenchyme of the vestibular lamina distal part (Figure 5D) and the diastema mesenchyme beneath the oral epithelium (Figure 5E). *Klk4* was not expressed in the molar region but was expressed in the buccal vestibular mesenchyme, which is outside of the tooth germ, non-odontogenic region (Figure 5F). *Gfra2* was expressed in the diastema of the distal side of the incisor vestibular lamina (Figure 5G). *Gfra2* was strongly and broadly expressed in the diastema mesenchyme region (Figure 5H). In the molar region, *Gfra2* expression was not observed (Figure 5I). *Cxcl14* expression pattern in the incisor region is similar to *Gfra2* and *Klk4* (Figure 5J). In the diastema, *Cxcl14* was observed beneath the oral epithelium (Figure 5K). In the molar region, *Cxcl14* is expressed in the buccal vestibular mesenchyme of the molar tooth germ (Figure 5L) similar to *Klk4*. *Cdh2* was expressed in whole oral epithelium of the embryo dental arch (Figures 5M–O). *Cdh2* was also expressed in the diastema mesenchyme (Figure 5N); however, it was not in the mesenchyme of the odontogenic regions (Figures 5M,O). In order to validate the *in situ* hybridization results of the selected genes, RT-qPCR was performed and normalized to the housekeeping gene *Gapdh*. All genes were significantly downregulated in the odontogenic region (molar) compared with the non-odontogenic region (diastema) (Supplementary Figure 4).

## Discussion

Studies on tooth regeneration conducted over the past 40 years have comprised two flows. One focused on epithelial–mesenchymal interaction in the odontogenic region. The others focused on creating a supernumerary tooth in the non-odontogenic region. Both research trends have been based on tooth development studies. Many signal pathways involved in tooth formation have been elucidated, and studies have been conducted to regenerate tooth with regulations of signal pathways. With the remarkable development of NGS technology, these studies are facing a new turning point. The latest research trend of the first flow is a study that differentiates induced pluripotent stem cells (iPSC) into dental epithelium and dental mesenchyme, respectively, and fuses them to induce epithelial–mesenchymal interaction (Zheng et al., 2019; Zhang and Yelick, 2021). It is accompanied by sophisticated differentiation regulation while confirming the degree of differentiation of iPSCs through transcriptome analysis as well as revealing single-cell sequencing results for each stage of tooth development using NGS. However, in the second flow, there has not been any published research incorporating NGS yet, and even the sequencing database of the diastema to compare with the odontogenic region has not been published.

This is the first study comparing RNA-Seq datasets of the odontogenic region and the diastema region. All sequencing datasets were uploaded to the NCBI Sequence Read Archive (Lee, 2021a), and metadata were published on Figshare (Lee, 2021b). PCA, DEG analysis, GO term analysis, and time series analysis with GO analysis were performed using these RNA-seq datasets. Through the PCA and heatmap of the entire datasets, it was confirmed that the biological replicates for each group were not biased and categorized well. In DEG analysis, the number of genes upregulated in the diastema region was 10 times higher at the dental lamina stage (E12.5) of tooth development, whereas the complex gene expression in the molar region increased as development progressed. It can be inferred that the region of more complex gene expression is converted from diastema to molar between E12.5 and E15.5, and in diastema, the diversity of gene expression decreases depending on the stage. In GO term analysis, terms related to mesenchyme development, cell fate commitment, cartilage and bone development, and extracellular matrix were mainly found in E12.5. Among those terms, ossification was shown to have the highest frequency clearly. In addition, expression patterns of genes belonging to embryonic organ development and DNA-binding transcription activator activity, which are development-related GO terms, were confirmed to be upregulated in diastema at E12.5 (Supplementary Figure 3). In E15.5, tissue development-related terms that were upregulated in diastema were shifted to the molar region, and the Wnt signaling pathway was upregulated. Also, the term odontogenesis first appeared. In E17.5, mRNA splicing-related terms were ranked higher. In general, RNA splicing is actively conducted when plenty of gene expression occurs, and alternative splicing has important physiological functions in different developmental processes (Baralle and Giudice, 2017). Some enamel matrix proteins are multi-cistronic, and in particular, AmelX requires alternative splicing to be operated in a very sophisticated and conserved manner (Simmer et al., 1994; Cho et al., 2014). It can be inferred that active splicing is required to form the tooth matrix at the E17.5 molar. On the other hand, in E15.5 and E17.5 of the diastema region, muscle development terms were mainly found. Throughout this interpretation, a limitation of this study can be raised that the gene expression analysis at the E12.5 stage may not be considered for early tissue determination in the developmental context, when considering the GO terms related to skeletal and cartilage lineage development at the E12.5 diastema region. Therefore, the desired time series analysis would provide useful insight into the tooth development study.

The time series analysis method of the RNA-seq data was already introduced; “The time-dependent changes in gene expression in biological systems can be analyzed by accounting for the dependencies of expression patterns across time points (Oh et al., 2013).” As the development progresses in the diastema and molar regions, the gene expression patterns change in opposite directions or move only in one region can be compared. With this approach, through time series analysis, the genes that turn on or off tooth formation can be screened. Especially, the genes involved in the tooth bud formation can be distinguished by comparing the gene expression patterns in the odontogenic and non-odontogenic regions in E12.5. Among the genes upregulated in the diastema and downregulated in the incisor and molar, genes that function as a switch to lose tooth-forming capacity during dental arch development and to remain in rudiment will be included. The candidate gene list derived from time series analysis of TS E12.5 is presented in Table 2.

*In situ* hybridization was performed to verify the expression propensity of some of the genes showing this expression pattern. Genes whose function has never been identified in tooth development were selected. The common pattern of the five selected genes was that the diastema mesenchyme showed stronger expression than the incisor and molar region mesenchyme. In addition, expression in the diastema mesenchyme was shown to lead to the distal side of the vestibular lamina near the incisor.

This study of RNA-Seq data including the non-odontogenic region presented a new milestone in one axis of tooth regeneration research, which has not yet been applied with NGS information. Time series analysis of the odontogenic/non-odontogenic region can be used in conjunction with DEG analysis and enables more specific analysis spatiotemporally than DEG analysis alone. The provided candidate gene list of TS E12.5 would present another milestone. Through gain-of-function and loss-of-function studies using candidates of tooth formation switch genes found in the diastema, it will be feasible to conduct unraveled research to generate tooth at non-odontogenic region or stop tooth development at desired positions.

## Data Availability Statement

The datasets presented in this study can be found in online repositories. The names of the repository/repositories and accession number(s) can be found below: https://www.ncbi.nlm.nih.gov/SRA/SRP308455 and https://doi.org/10.6084/m9.figshare.c.5448027.

## Ethics Statement

The animal study was reviewed and approved by the Yonsei University Health System, Intramural Animal Care and Use Committee (Approval No. 2020-0146).

## Author Contributions

D-JL and H-SJ contributed to the conception and design of the study. D-JL and H-YK analyzed the data. D-JL and S-JL collected the samples and performed the experiments. D-JL drafted the manuscript. H-SJ conceived and supervised the project. All authors contributed to the article and approved the submitted version.

## Conflict of Interest

H-YK was employed by company NGeneS Inc. The remaining authors declare that the research was conducted in the absence of any commercial or financial relationships that could be construed as a potential conflict of interest.

## Publisher’s Note

All claims expressed in this article are solely those of the authors and do not necessarily represent those of their affiliated organizations, or those of the publisher, the editors and the reviewers. Any product that may be evaluated in this article, or claim that may be made by its manufacturer, is not guaranteed or endorsed by the publisher.
