# Supplementary material for: Spatiotemporal Changes in Transcriptome of Odontogenic and Non-odontogenic Regions in the Dental Arch of Mus musculus
Source: Front Cell Dev Biol. 2021 Oct 14;9:723326. doi: 10.3389/fcell.2021.723326 (PMC8551760; doi:10.3389/fcell.2021.723326)
Supplement: Supplementary file 1 [file Data_Sheet_1.PDF]

Supplementary material

No	Sample ID	Concentration (ng/μℓ)	Volume (μℓ)	Quantity (μg)	Purity	
					RIN	28s/18s
1	E12.5_incisor_Set1	25.42	12	0.305	9.4	1.4
2	E12.5_incisor_Set2	101.00	14	1.414	9.8	1.6
3	E12.5_incisor_Set3	118.00	14	1.652	9.3	1.3
4	E12.5_diastema_Set1	125.00	8	1.000	9.7	1.6
5	E12.5_diastema_Set2	45.00	14	0.630	9.9	1.6
6	E12.5_diastema_Set3	75.00	14	1.050	9.8	1.6
7	E12.5_molar_Set1	68.33	15	1.025	9.5	1.3
8	E12.5_molar_Set2	74.00	14	1.036	10.0	1.6
9	E12.5_molar_Set3	134.00	14	1.876	9.7	1.4
10	E15.5_diastema_Set1	250.00	16	4.000	9.8	1.7
11	E15.5_diastema_Set2	241.00	14	3.374	9.4	1.5
12	E15.5_diastema_Set3	234.00	13	3.042	9.8	1.9
13	E15.5_molar_Set1	285.00	16	4.560	10.0	1.7
14	E15.5_molar_Set2	279.00	14	3.906	9.7	1.6
15	E15.5_molar_Set3	235.00	15	3.525	9.8	1.7
16	E17.5_diastema_Set1	168.38	8	1.347	8.9	1.6
17	E17.5_diastema_Set2	217.00	14	3.038	9.0	1.6
18	E17.5_diastema_Set3	195.00	16	3.120	9.4	1.5
19	E17.5_molar_Set1	164.00	8	1.312	9.6	1.7
20	E17.5_molar_Set2	327.00	14	4.578	9.7	1.8
21	E17.5_molar_Set3	180.00	15	2.700	9.8	1.7

Supplementary Table 1 | QC data of RNA preparation

Gene		Forward (5’-3’)	Reverse (5’-3’)	Product size
RT-qPCR	Zfp618	GTCGAACCAGTCGGGAAAA	TCTGCTGCTGCTGCCTTG	141
	Gfra2	ACGAAACCCTCCGCTCTTTG	AGCGGCAGTCATACAATGGG	243
	Klk4	GTGTCAGCAGCCGGATCATA	CCAAGACTCCCGAGCAGAAA	105
	Cxcl14	CTGAGTCACCGAGTGGTTCTG	CGGCTTGGTTCTCGGTTTCA	228
	Cdh2	CGCAGTCTTACCGAAGGATGT	TCTCACAGCATACACCGTGC	148
In situ hybridization	Zfp618	TCGGAGTCGGTATTCAGGGA	TCGGAACGTCTGAGTTTGGG	667
	Gfra2	TTAGACGAAACCCTCCGCTC	ACAGTTGGCGTGGAAGTCTG	789
	Klk4	CTGCTCGGGAGTCTTGGTG	CTGAGGTGGTACACAGGGTC	402
	Cxcl14	GAGTCACCGAGTGGTTCTGCAT	CTTCGTAGACCCTGCGCTTC	582
	Cdh2	ATGTGCCGGATAGCGGGAG	AGTTGATTGGCGGGATGACC	502

Supplementary Table 2 | Primer sequences for RT-qPCR and *in situ* hybridization

No	Sample ID	Raw (Adapter trimmed)	Filtered	Mapped	Uniquely* Mapped	READ 1** / READ 2	Strand(+)*** / Strand(-)	Splice****
1	E12.5_incisor_Set1	48,715,568	46,449,704<br>95.3%	40,705,265<br>87.6%	38,878,314<br>83.7%	21,038,358<br>17,839,956	19,398,692<br>19,479,622	15,550,402<br>33.5%
2	E12.5_incisor_Set2	47,362,550	45,972,810<br>97.1%	40,053,846<br>87.1%	38,337,609<br>83.4%	20,924,805<br>17,412,804	19,130,703<br>19,206,906	15,768,685<br>34.3%
3	E12.5_incisor_Set3	67,657,256	65,783,410<br>97.2%	56,441,728<br>85.8%	53,708,152<br>81.6%	29,802,157<br>23,905,995	26,783,768<br>26,924,384	22,692,644<br>34.5%
4	E12.5_diastema_Set1	57,962,590	55,705,366<br>96.1%	49,454,256<br>88.8%	47,761,329<br>85.7%	25,554,348<br>22,206,981	23,858,589<br>23,902,740	20,286,257<br>36.4%
5	E12.5_diastema_Set2	40,265,360	38,914,678<br>96.6%	33,813,188<br>86.9%	32,360,700<br>83.2%	17,641,966<br>14,718,734	16,153,004<br>16,207,696	13,157,577<br>33.8%
6	E12.5_diastema_Set3	70,319,504	68,340,666<br>97.2%	57,387,493<br>84.0%	54,779,372<br>80.2%	31,063,031<br>23,716,341	27,316,225<br>27,463,147	22,074,764<br>32.3%
7	E12.5_molar_Set1	56,314,710	53,430,272<br>94.9%	46,438,486<br>86.9%	44,346,422<br>83.0%	24,104,280<br>20,242,142	22,112,809<br>22,233,613	17,287,676<br>32.4%
8	E12.5_molar_Set2	41,484,366	40,165,268<br>96.8%	34,541,334<br>86.0%	33,037,716<br>82.3%	18,201,981<br>14,835,735	16,475,137<br>16,562,579	13,329,552<br>33.2%
9	E12.5_molar_Set3	74,032,748	72,230,840<br>97.6%	62,549,392<br>86.6%	59,830,291<br>82.8%	32,964,214<br>26,866,077	29,857,510<br>29,972,781	24,985,344<br>34.6%
10	E15.5_diastema_Set1	68,201,930	65,578,424<br>96.2%	58,127,043<br>88.6%	56,110,249<br>85.6%	30,071,632<br>26,038,617	28,022,154<br>28,088,095	23,535,501<br>35.9%
11	E15.5_diastema_Set2	45,951,920	44,552,930<br>97.0%	39,043,651<br>87.6%	37,273,622<br>83.7%	20,250,115<br>17,023,507	18,627,610<br>18,646,012	16,244,147<br>36.5%
12	E15.5_diastema_Set3	47,605,924	46,449,162<br>97.6%	40,104,295<br>86.3%	38,411,415<br>82.7%	21,041,237<br>17,370,178	19,159,787<br>19,251,628	15,426,257<br>33.2%
13	E15.5_molar_Set1	72,807,624	69,933,160<br>96.1%	61,992,858<br>88.6%	59,914,986<br>85.7%	32,093,881<br>27,821,105	29,932,211<br>29,982,775	25,042,822<br>35.8%
14	E15.5_molar_Set2	40,305,860	39,182,120<br>97.2%	34,555,595<br>88.2%	33,216,574<br>84.8%	17,934,265<br>15,282,309	16,593,997<br>16,622,577	14,032,009<br>35.8%
15	E15.5_molar_Set3	57,631,506	56,401,312<br>97.9%	48,252,320<br>85.6%	46,314,813<br>82.1%	25,339,023<br>20,975,790	23,070,976<br>23,243,837	18,716,158<br>33.2%

continued

16	E17.5_diastema_Set1	67,759,816	64,452,330<br>95.1%	56,786,196<br>88.1%	54,481,654<br>84.5%	29,361,607<br>25,120,047	27,219,514<br>27,262,140	23,484,004<br>36.4%
17	E17.5_diastema_Set2	37,430,834	36,154,878<br>96.6%	31,314,258<br>86.6%	29,935,593<br>82.8%	16,268,121<br>13,667,472	14,963,718<br>14,971,875	12,545,996<br>34.7%
18	E17.5_diastema_Set3	53,086,272	51,944,572<br>97.8%	45,225,300<br>87.1%	43,162,099<br>83.1%	23,502,047<br>19,660,052	21,580,320<br>21,581,779	18,302,097<br>35.2%
19	E17.5_molar_Set1	49,579,086	47,172,774<br>95.1%	41,316,457<br>87.6%	39,778,971<br>84.3%	21,519,035<br>18,259,936	19,872,971<br>19,906,000	17,265,023<br>36.6%
20	E17.5_molar_Set2	50,722,064	49,153,708<br>96.9%	41,862,482<br>85.2%	40,327,864<br>82.0%	22,309,490<br>18,018,374	20,128,714<br>20,199,150	16,565,283<br>33.7%
21	E17.5_molar_Set3	53,950,508	52,679,148<br>97.6%	45,403,135<br>86.2%	43,663,884<br>82.9%	23,947,002<br>19,716,882	21,778,928<br>21,884,956	18,426,622<br>35.0%
	Total	1,149,147,996	1,110,647,532<br>96.65%	965,368,578<br>84.01%	925,631,629<br>80.55%			384,718,820<br>33.48%

Supplementary Table 3 | Summary statistics of RNA-seq data

*	Uniquely Mapped	reads mapped to exactly one location within the reference genome
**	READ1/READ2	reads on Forward / reads on Reverse
***	Strand(+)/Strand(-)	DNA sense / DNA antisense
****	Splice	mapped reads on splicing site

E12.5_incisor_Set1

Category	Genome		Genes	
	Reads Number	%	Reads Number	%
Total Reads	46,449,704	100.00	46,449,704	100.00
Mapped Reads	40,705,265	87.63	40,734,204	87.70
Properly paired*	34,101,544	73.42	39,845,992	85.78

E12.5_incisor_Set2

Category	Genome		Genes	
	Reads Number	%	Reads Number	%
Total Reads	45,972,810	100.00	45,972,810	100.00
Mapped Reads	40,053,846	87.13	40,972,748	89.12
Properly paired*	33,328,074	72.50	40,161,072	87.36

E12.5_incisor_Set3

Category	Genome		Genes	
	Reads Number	%	Reads Number	%
Total Reads	65,783,410	100.00	65,783,410	100.00
Mapped Reads	56,441,728	85.80	58,730,667	89.28
Properly paired*	45,829,610	69.67	57,608,208	87.57

E12.5_diastema_Set1

Total Reads	55,705,366	100.00	55,705,366	100.00
Mapped Reads	49,454,256	88.78	49,960,602	89.69
Properly paired	42,175,588	75.71	49,007,730	87.98

E12.5_diastema_Set2

Total Reads	38,914,678	100.00	38,914,678	100.00
Mapped Reads	33,813,188	86.89	34,564,591	88.82
Properly paired	28,202,780	72.47	33,907,244	87.13

E12.5_diastema_Set3

Total Reads	68,340,666	100.00	68,340,666	100.00
Mapped Reads	57,387,493	83.97	61,358,817	89.78
Properly paired	45,473,744	66.54	60,229,754	88.13

E12.5_molar_Set1

Total Reads	53,430,272	100.00	53,430,272	100.00
Mapped Reads	46,438,486	86.91	46,367,353	86.78
Properly paired	38,619,580	72.28	45,307,556	84.80

E12.5_molar_Set2

Total Reads	40,165,268	100.00	40,165,268	100.00
Mapped Reads	34,541,334	86.00	35,746,168	89.00
Properly paired	28,368,764	70.63	35,029,168	87.21

E12.5_molar_Set3

Total Reads	72,230,840	100.00	72,230,840	100.00
Mapped Reads	62,549,392	86.60	64,619,353	89.46
Properly paired	51,544,286	71.36	63,363,120	87.72

E15.5_diastema_Set1

Total Reads	65,578,424	100.00	65,578,424	100.00
Mapped Reads	58,127,043	88.64	58,451,766	89.13
Properly paired	49,246,130	75.10	57,187,746	87.21

E15.5_diastema_Set2

Total Reads	44,552,930	100.00	44,552,930	100.00
Mapped Reads	39,043,651	87.63	40,443,846	90.78
Properly paired	32,416,126	72.76	39,822,998	89.38

E15.5_diastema_Set3

Total Reads	46,449,162	100.00	46,449,162	100.00
Mapped Reads	40,104,295	86.34	40,757,878	87.75
Properly paired	33,257,798	71.60	40,015,284	86.15

Total Reads	69,933,160	100.00	69,933,160	100.00
Mapped Reads	61,992,858	88.65	62,289,602	89.07
Properly paired	52,789,374	75.49	60,948,288	87.15

Total Reads	39,182,120	100.00	39,182,120	100.00
Mapped Reads	34,555,595	88.19	35,267,282	90.01
Properly paired	29,188,058	74.49	34,604,804	88.32

Total Reads	56,401,312	100.00	56,401,312	100.00
Mapped Reads	48,252,320	85.55	48,415,238	85.84
Properly paired	40,142,360	71.17	47,529,236	84.27

continued

E17.5_diastema_Set1

Total Reads	64,452,330	100.00	64,452,330	100.00
Mapped Reads	56,786,196	88.11	58,080,749	90.11
Properly paired	47,450,652	73.62	56,882,798	88.26

E17.5_diastema_Set2

36,154,878	100.00	36,154,878	100.00
31,314,258	86.61	32,552,421	90.04
26,043,910	72.03	32,005,344	88.52

E17.5_diastema_Set3

51,944,572	100.00	51,944,572	100.00
45,225,300	87.06	46,205,389	88.95
37,737,786	72.65	45,415,206	87.43

E17.5_molar_Set1

Total Reads	47,172,774	100.00	47,172,774	100.00
Mapped Reads	41,316,457	87.59	42,559,211	90.22
Properly paired	34,666,286	73.49	41,779,360	88.57

E17.5_molar_Set2

49,153,708	100.00	49,153,708	100.00
41,862,482	85.17	43,352,755	88.20
34,348,512	69.88	42,551,446	86.57

E17.5_molar_Set3

52,679,148	100.00	52,679,148	100.00
45,403,135	86.19	46,542,900	88.35
37,898,544	71.94	45,753,282	86.85

Supplementary Table 4 | Statistics of mapping to genome and genes

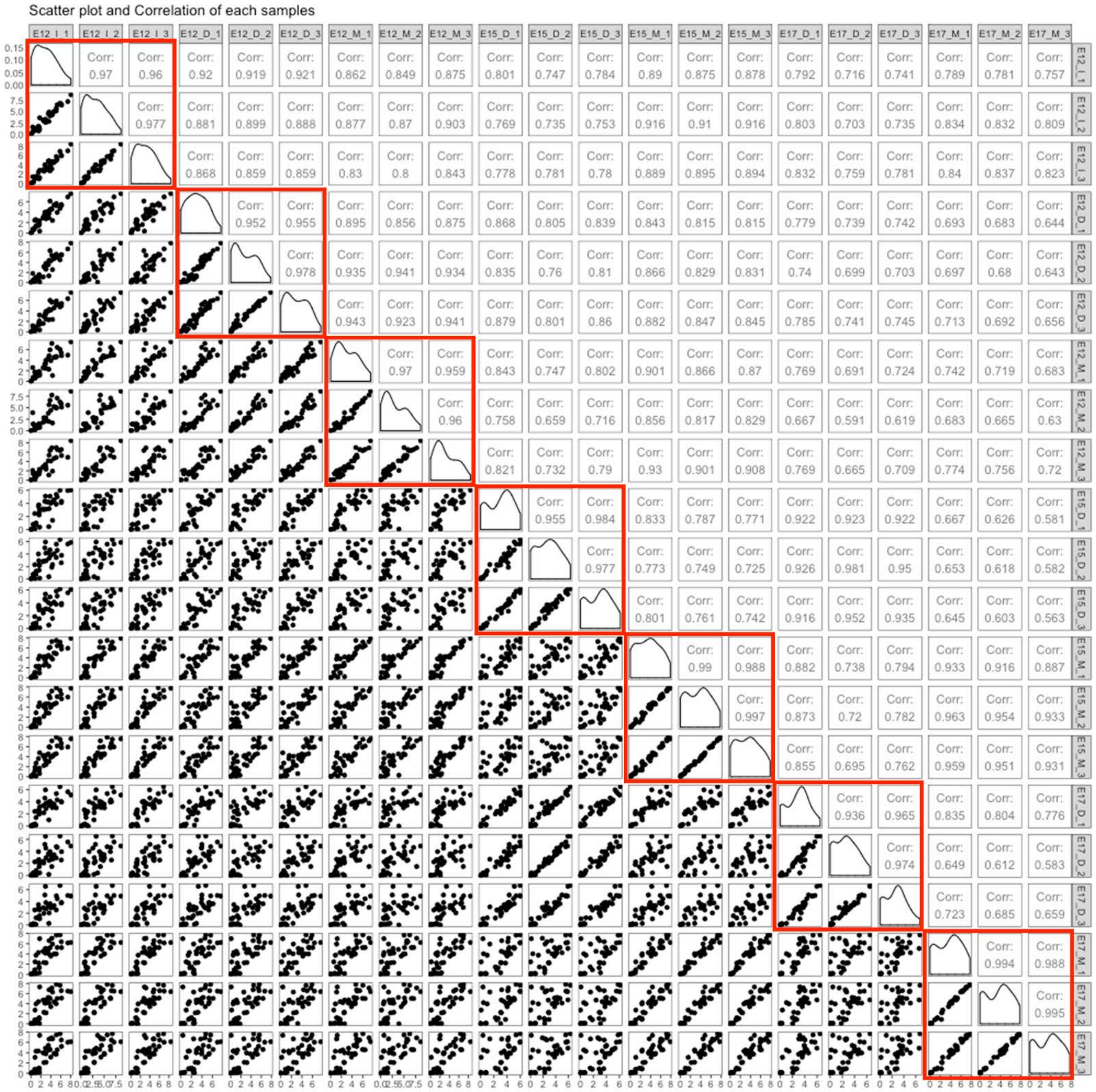

Supplementary Figure 1 | Scatter plot and correlation of each sample

Pearson’s correlation values in each experimental group are higher than 0.95 (red boxes); only the correlation value of E17.5 diastema set1 and set 2 was found to be 0.936.

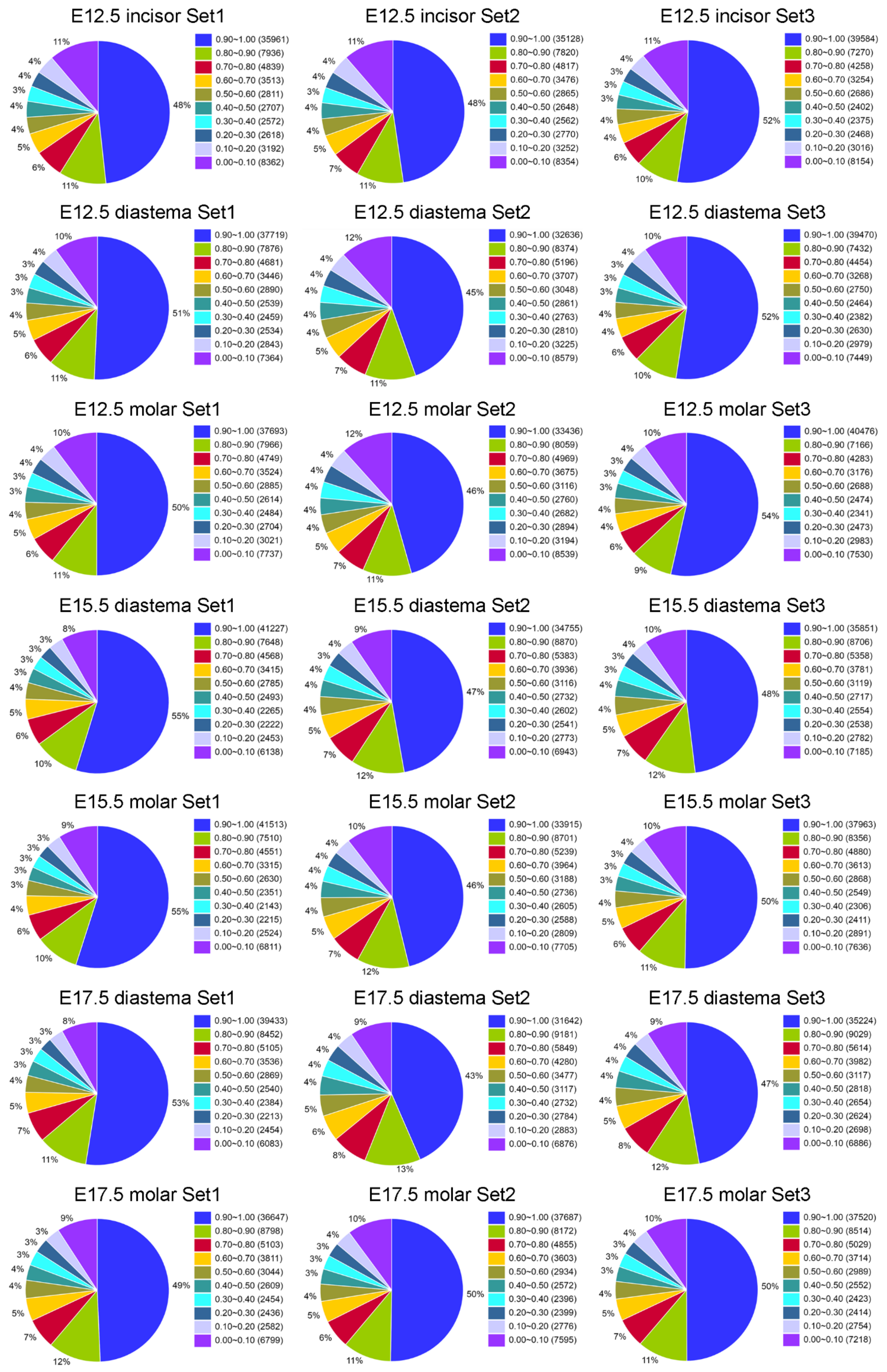
E12.5 incisor Set1
48% 11% 6% 5% 4% 4% 3% 4% 4% 11%
0.90~1.00 (35961)
0.80~0.90 (7936)
0.70~0.80 (4839)
0.60~0.70 (3513)
0.50~0.60 (2811)
0.40~0.50 (2707)
0.30~0.40 (2572)
0.20~0.30 (2618)
0.10~0.20 (3192)
0.00~0.10 (8362)
E12.5 incisor Set2
48% 11% 7% 5% 4% 4% 3% 4% 4% 11%
0.90~1.00 (35128)
0.80~0.90 (7820)
0.70~0.80 (4817)
0.60~0.70 (3476)
0.50~0.60 (2865)
0.40~0.50 (2648)
0.30~0.40 (2562)
0.20~0.30 (2770)
0.10~0.20 (3252)
0.00~0.10 (8354)
E12.5 incisor Set3
52% 10% 6% 4% 4% 3% 3% 3% 4% 11%
0.90~1.00 (39584)
0.80~0.90 (7270)
0.70~0.80 (4258)
0.60~0.70 (3254)
0.50~0.60 (2686)
0.40~0.50 (2402)
0.30~0.40 (2375)
0.20~0.30 (2468)
0.10~0.20 (3016)
0.00~0.10 (8154)
E12.5 diastema Set1
51% 11% 6% 5% 4% 3% 3% 3% 4% 10%
0.90~1.00 (37719)
0.80~0.90 (7876)
0.70~0.80 (4681)
0.60~0.70 (3446)
0.50~0.60 (2890)
0.40~0.50 (2539)
0.30~0.40 (2459)
0.20~0.30 (2534)
0.10~0.20 (2843)
0.00~0.10 (7364)
E12.5 diastema Set2
45% 11% 7% 5% 4% 4% 4% 4% 4% 12%
0.90~1.00 (32636)
0.80~0.90 (8374)
0.70~0.80 (5196)
0.60~0.70 (3707)
0.50~0.60 (3048)
0.40~0.50 (2861)
0.30~0.40 (2763)
0.20~0.30 (2810)
0.10~0.20 (3225)
0.00~0.10 (8579)
E12.5 diastema Set3
52% 10% 6% 4% 4% 3% 3% 3% 4% 10%
0.90~1.00 (39470)
0.80~0.90 (7432)
0.70~0.80 (4454)
0.60~0.70 (3268)
0.50~0.60 (2750)
0.40~0.50 (2464)
0.30~0.40 (2382)
0.20~0.30 (2630)
0.10~0.20 (2979)
0.00~0.10 (7449)
E12.5 molar Set1
50% 11% 6% 5% 4% 3% 3% 4% 4% 10%
0.90~1.00 (37693)
0.80~0.90 (7966)
0.70~0.80 (4749)
0.60~0.70 (3524)
0.50~0.60 (2885)
0.40~0.50 (2614)
0.30~0.40 (2484)
0.20~0.30 (2704)
0.10~0.20 (3021)
0.00~0.10 (7737)
E12.5 molar Set2
46% 11% 7% 5% 4% 4% 4% 4% 4% 12%
0.90~1.00 (33436)
0.80~0.90 (8059)
0.70~0.80 (4969)
0.60~0.70 (3675)
0.50~0.60 (3116)
0.40~0.50 (2760)
0.30~0.40 (2682)
0.20~0.30 (2894)
0.10~0.20 (3194)
0.00~0.10 (8539)
E12.5 molar Set3
54% 9% 6% 4% 4% 3% 3% 3% 4% 10%
0.90~1.00 (40476)
0.80~0.90 (7166)
0.70~0.80 (4283)
0.60~0.70 (3176)
0.50~0.60 (2688)
0.40~0.50 (2474)
0.30~0.40 (2341)
0.20~0.30 (2473)
0.10~0.20 (2983)
0.00~0.10 (7530)
E15.5 diastema Set1
55% 10% 6% 5% 4% 3% 3% 3% 3% 8%
0.90~1.00 (41227)
0.80~0.90 (7648)
0.70~0.80 (4568)
0.60~0.70 (3415)
0.50~0.60 (2785)
0.40~0.50 (2493)
0.30~0.40 (2265)
0.20~0.30 (2222)
0.10~0.20 (2453)
0.00~0.10 (6138)
E15.5 diastema Set2
47% 12% 7% 5% 4% 4% 4% 3% 4% 9%
0.90~1.00 (34755)
0.80~0.90 (8870)
0.70~0.80 (5383)
0.60~0.70 (3936)
0.50~0.60 (3116)
0.40~0.50 (2732)
0.30~0.40 (2602)
0.20~0.30 (2541)
0.10~0.20 (2773)
0.00~0.10 (6943)
E15.5 diastema Set3
48% 12% 7% 5% 4% 4% 3% 3% 4% 10%
0.90~1.00 (35851)
0.80~0.90 (8706)
0.70~0.80 (5358)
0.60~0.70 (3781)
0.50~0.60 (3119)
0.40~0.50 (2717)
0.30~0.40 (2554)
0.20~0.30 (2538)
0.10~0.20 (2782)
0.00~0.10 (7185)
E15.5 molar Set1
55% 10% 6% 4% 3% 3% 3% 3% 3% 9%
0.90~1.00 (41513)
0.80~0.90 (7510)
0.70~0.80 (4551)
0.60~0.70 (3315)
0.50~0.60 (2630)
0.40~0.50 (2351)
0.30~0.40 (2143)
0.20~0.30 (2215)
0.10~0.20 (2524)
0.00~0.10 (6811)
E15.5 molar Set2
46% 12% 7% 5% 4% 4% 4% 4% 4% 10%
0.90~1.00 (33915)
0.80~0.90 (8701)
0.70~0.80 (5239)
0.60~0.70 (3964)
0.50~0.60 (3188)
0.40~0.50 (2736)
0.30~0.40 (2605)
0.20~0.30 (2588)
0.10~0.20 (2809)
0.00~0.10 (7705)
E15.5 molar Set3
50% 11% 6% 5% 4% 3% 3% 3% 4% 10%
0.90~1.00 (37963)
0.80~0.90 (8356)
0.70~0.80 (4880)
0.60~0.70 (3613)
0.50~0.60 (2868)
0.40~0.50 (2549)
0.30~0.40 (2306)
0.20~0.30 (2411)
0.10~0.20 (2891)
0.00~0.10 (7636)
E17.5 diastema Set1
53% 11% 7% 5% 4% 3% 3% 3% 3% 8%
0.90~1.00 (39433)
0.80~0.90 (8452)
0.70~0.80 (5105)
0.60~0.70 (3536)
0.50~0.60 (2869)
0.40~0.50 (2540)
0.30~0.40 (2384)
0.20~0.30 (2213)
0.10~0.20 (2454)
0.00~0.10 (6083)
E17.5 diastema Set2
43% 13% 8% 6% 5% 4% 4% 4% 4% 9%
0.90~1.00 (31642)
0.80~0.90 (9181)
0.70~0.80 (5849)
0.60~0.70 (4280)
0.50~0.60 (3477)
0.40~0.50 (3117)
0.30~0.40 (2732)
0.20~0.30 (2784)
0.10~0.20 (2883)
0.00~0.10 (6876)
E17.5 diastema Set3
47% 12% 8% 5% 4% 4% 4% 4% 4% 9%
0.90~1.00 (35224)
0.80~0.90 (9029)
0.70~0.80 (5614)
0.60~0.70 (3982)
0.50~0.60 (3177)
0.40~0.50 (2818)
0.30~0.40 (2654)
0.20~0.30 (2624)
0.10~0.20 (2698)
0.00~0.10 (6886)
E17.5 molar Set1
49% 12% 7% 5% 4% 4% 3% 3% 3% 9%
0.90~1.00 (36647)
0.80~0.90 (8798)
0.70~0.80 (5103)
0.60~0.70 (3811)
0.50~0.60 (3044)
0.40~0.50 (2609)
0.30~0.40 (2454)
0.20~0.30 (2436)
0.10~0.20 (2582)
0.00~0.10 (6799)
E17.5 molar Set2
50% 11% 6% 5% 4% 3% 3% 3% 4% 10%
0.90~1.00 (37687)
0.80~0.90 (8172)
0.70~0.80 (4855)
0.60~0.70 (3603)
0.50~0.60 (2934)
0.40~0.50 (2572)
0.30~0.40 (2396)
0.20~0.30 (2399)
0.10~0.20 (2776)
0.00~0.10 (7595)
E17.5 molar Set3
50% 11% 7% 5% 4% 3% 3% 3% 4% 10%
0.90~1.00 (37520)
0.80~0.90 (8514)
0.70~0.80 (5029)
0.60~0.70 (3714)
0.50~0.60 (2989)
0.40~0.50 (2552)
0.30~0.40 (2423)
0.20~0.30 (2414)
0.10~0.20 (2754)
0.00~0.10 (7218)

Supplementary Figure 2 | Average coverage of the reads aligned to mRNA

The coverage range was divided by 0.1 and from 0 to 1. The number of reads for the range covering the mRNA sequence references is presented in the legends of each sample. Reads with a coverage range of 0.9 to 1.0 accounted for 45%–55% in almost all samples.

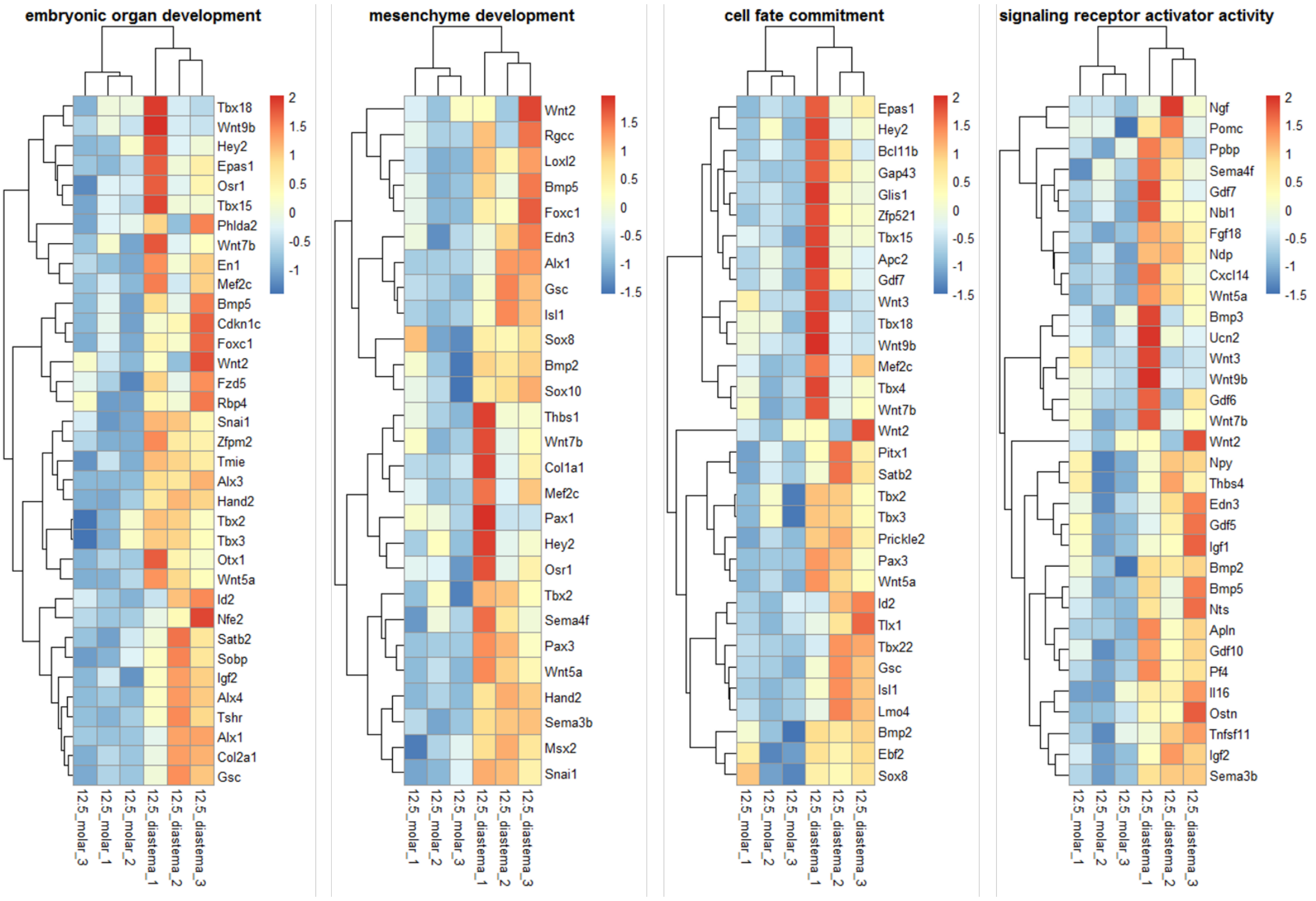

Supplementary Figure 3 | E12.5 mRNA expression by GO term in molar and diastema region

Among the GO terms ranked higher in E12.5 diastema and molar, only genes belonging to terms related to organogenesis and tooth development were expressed as heatmaps.

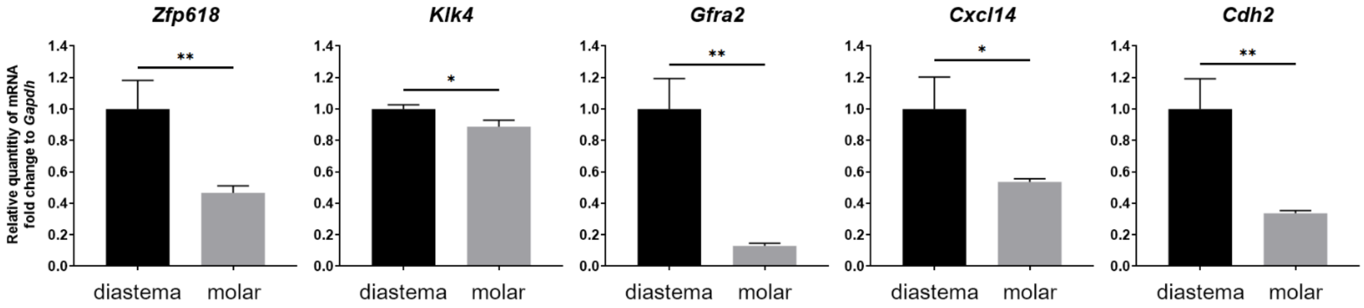

Supplementary Figure 4 | RT-qPCR results of E12.5 non-odontogenic and odontogenic regions

Related quantities of mRNA of selected genes were analyzed by RT-qPCR. * p<0.05, ** p<0.01.
